# Has Covid-19 permanently changed online purchasing behavior?

**DOI:** 10.1140/epjds/s13688-022-00375-1

**Published:** 2023-01-16

**Authors:** Hiroyasu Inoue, Yasuyuki Todo

**Affiliations:** 1 Graduate School of Information Science, University of Hyogo, 650-0047 Kobe, Japan; 2 Japan Science and Technology Agency, 332-0012 Saitama, Japan; 3 RIKEN Center for Computational Science, Wako, 351-0198 Saitama, Japan; 4 Graduate School of Economics, Waseda University, 169-8050 Tokyo, Japan; 5 Research Institute of Economy, Trade and Industry, 100-0013 Tokyo, Japan

**Keywords:** Consumer behavior, Online purchasing, Online shopping, Covid-19, State of emergency, Stay at home, BtoC

## Abstract

This study examines how the COVID-19 pandemic has affected online purchasing behavior using data from a major online shopping platform in Japan. We focus on the effect of two measures of the pandemic, i.e., the number of positive COVID-19 cases and state declarations of emergency to mitigate the pandemic. We find that both measures promoted online purchases at the beginning of the pandemic, but in later periods, their effect faded. In addition, online purchases returned to normal after states of emergency ended, and the overall time trend in online purchases excluding the effects of the two measures was stable during the first two years of the pandemic. These results suggest that the effect of the pandemic on online purchasing behavior is temporary and will not persist after the pandemic.

**Supplementary Information:**

The online version contains supplementary material available at 10.1140/epjds/s13688-022-00375-1.

## Introduction

The COVID-19 pandemic that emerged in early 2020 largely affected consumer behavior. Accordingly, a number of academic studies have examined the effect of the pandemic and associated policies on consumption from various perspectives. During this time, consumers purchased more essential products, such as medical and health products and foods, while total purchases decreased because of a decline in the purchasing of nonessential products [1–14]. One possible reason for the increase in the purchasing of essential products was “panic buying” and hoarding because consumers were afraid that the pandemic and government restrictions would result in a shortage of supplies [3, 10, 15, 16], sometimes stimulated by mass media [17]. Similar purchasing behavior has been observed during other disasters, such as Hurricane Katrina in 2005 [18] and the 2009 swine flu pandemic caused by the H1N1 influenza virus [19].

One strand of the literature has focused on the effect of the COVID-19 pandemic on online shopping. Because government restrictions, including lockdowns, often require or request that people stay home and businesses close down, consumers may shop in person less frequently and thus rely more on online shopping. In addition, even without any restrictions, consumers may stay home out of fear of infection. This phenomenon has been confirmed by several studies based on surveys in the US [20, 21], China [22], and Vietnam [23] and by a study based on online transaction data at the country level in France [24].

One remaining question regarding the effect of the pandemic on online shopping is whether the increase in online shopping will persist after the pandemic. In the early period of the pandemic, several authors predicted that the pandemic would lead to permanent changes in consumer behavior, including the increased usage of online shopping channels [16, 25]. In addition, it has been empirically found that consumers in the US plan to continue grocery shopping online after the pandemic [20] and that online shopping in Vietnam will continue while fears about the pandemic are sufficiently high [23].

On the one hand, these predictions are justified because participating in online shopping incurs initial costs such as setting up new accounts, learning how to use online shopping sites safely, and learning how to evaluate online products accurately [26]. Therefore, once consumers were forced by the pandemic to begin online shopping, they became likely to continue to shop online because of the benefits of doing so, such as easier information searching and lower transportation costs [25].

On the other hand, the continued use of online shopping is not guaranteed because shopping in physical stores, or offline shopping, is still beneficial. For example, offline shopping is often motivated by the desire for immediate possession and social interactions [25, 27]. Moreover, online shopping is not only useful but also associated with certain costs. Privacy and security are at risk when shopping online. Processing online information requires greater cognitive resources than does processing printed information [26]. If the net benefit of offline shopping is greater than that of online shopping, then consumers will rely on offline shopping, even after paying the initial costs of online shopping. Furthermore, although consumers often panic and overreact at the beginning of a large shock, over time, they learn to cope with the new situation and react less to the shock [24]. The effect of the COVID-19 pandemic on fear and anxiety has also been found to diminish over time [28]. Therefore, consumers who are accustomed to the pandemic may have weaker reactions to it than they did before and return to old habits in the long run [16].

Whether the effect of the COVID-19 pandemic on online purchasing behavior will persist into the future has rarely been tested empirically in the academic literature using data covering a long period of the pandemic [24]. One exceptional study [29] used large-scale data on credit card transactions in Japan in January and April 2019 and 2020. The study found that although the share of online shopping in total purchases increased during this period of the pandemic, the probability that nonusers of online shopping started online shopping after the pandemic began was the same as that in the prepandemic period. Accordingly, once the pandemic subsides, online purchases are predicted to return to prepandemic levels.

The present study revisits this question using data on the purchase amount on a major online shopping platform in Japan from January 2019 to October 2021. Specifically, we employ two measures of pandemic shocks: the number of positive COVID-19 cases and the declaration of a state of emergency to mitigate the effects of the pandemic. Then, we estimate the effect of each measure on online purchases on the platform using a two-way fixed-effects (TWFE) approach. Our approach is relevant because our sample period covers one year preceding the pandemic, several waves of COVID-19 cases, and several stages of states of emergency and because the level of pandemic shocks varied substantially across prefectures in Japan. In addition, we check whether the effect of these shocks diminished over time. Finally, to determine whether the effect of these shocks persisted, we examine how online purchases changed after each wave of COVID-19 cases and states of emergency and how the time trend in online purchases changed over time after excluding the direct effect of the shocks.

## Data

Our data are taken from three sources: Yahoo! Shopping, COVID-19 cases, and information on states of emergency. The details are described in the following subsections.

### Yahoo! Shopping

**Figure 1:**
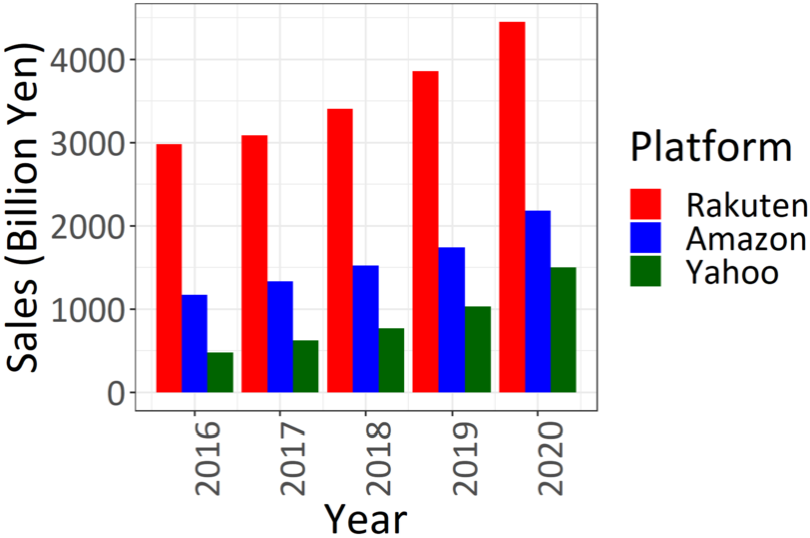
Annual Sales of the Top Three Online Platforms. The top three online platforms in Japan are Rakuten Group, Inc., Amazon Japan G.K., and Z Holdings (the parent company of Yahoo! Japan). We used all of the Amazon Japan sales since Amazon Japan does not publish online shopping sales alone. Data source: [30–32]

**Figure 2:**
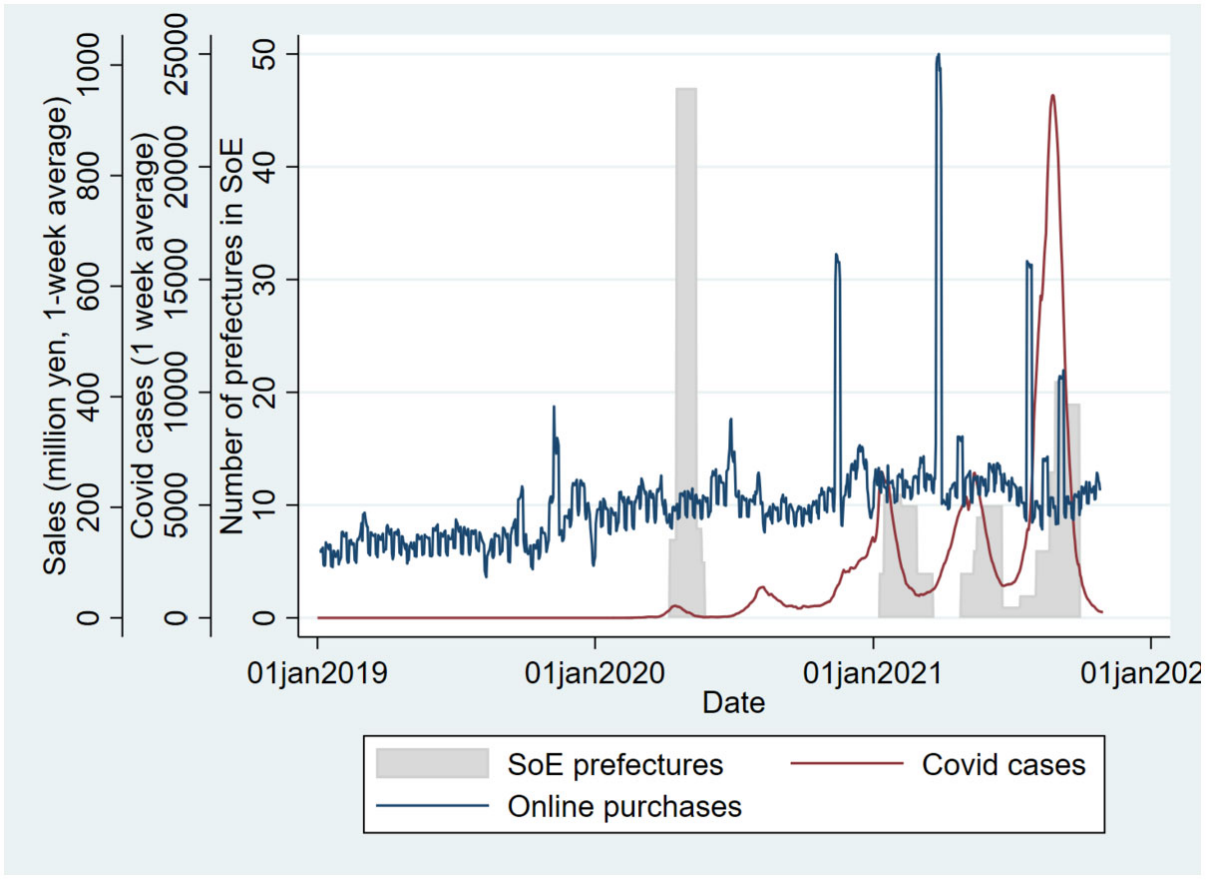
Changes in COVID-19 Cases and Online Purchases and Buyers over Time from January 1, 2019, to October 30, 2021. This figure shows the total number of daily positive COVID-19 cases in Japan averaged over one week (the red line), the number of prefectures in a state of emergency (SoE) due to COVID-19 (the shaded area), and the total number of daily purchases on the online shopping platform in Japan (the blue line). Data source: Ministry of Health, Labor and Welfare [33] and Cabinet Secretariat of Japan [34]

We utilize the daily data on the purchase amount and the number of buyers on an online shopping platform, Yahoo! Shopping (hereafter Yahoo). Yahoo is the third largest online shopping platform in Japan, following Amazon Japan and Rakuten, and its total sales in 2019 were 1501 billion Japanese yen, or approximately 13.7 billion US$ using the average exchange rate for that year [30] (Fig. 1). The number of buyers is defined as the number of “unique users,” not double-counting individuals who buy from the platform more than once in a day using the same internet protocol (IP) address. Our data cover the period from January 1, 2019, to October 30, 2021, and are aggregated at either the city- or prefecture–product level. The blue line in Fig. 2 shows the moving average of the total purchases for one week to avoid fluctuations due to days of the week. Notably, there are several spikes, most likely because of sale periods.

In each observation, when the number of buyers is quite small and individual buyers might thus be identified, the purchase amount and number of buyers are hidden due to Yahoo’s privacy policy. Therefore, among 1718 cities, wards, towns, and villages, which we broadly define as “cities,” in Japan, our city-level data include approximately 1100 data points each day.

**Figure 3:**
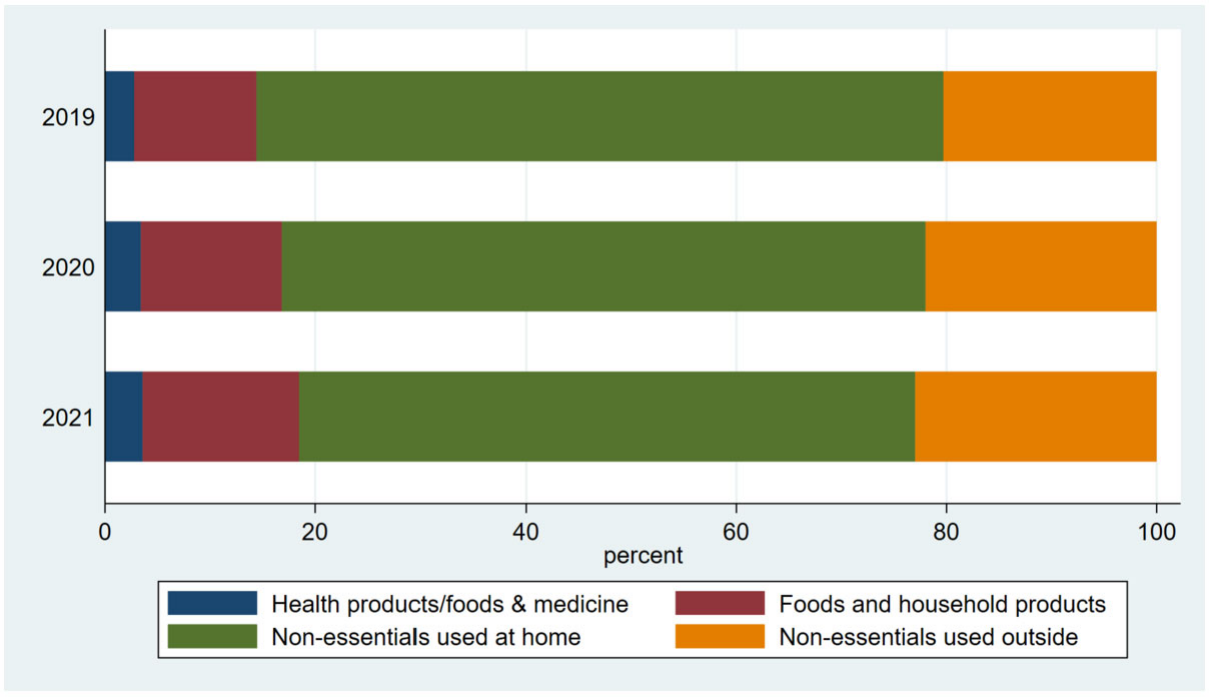
Share of the Purchase Amount in Each Year by Category. This figure shows the share of each of the four categories of the total purchase amount on the online shopping site in each year

In some of our analyses, we examine heterogeneity in the effect of the pandemic on online purchasing across products. For this purpose, we categorize products into four categories in the following way. Yahoo classifies products into 17 categories. Among them, the purchase amount of “gifts” and “housekeeping services” are often missing, possibly because of their small numbers, and purchases of “videos and music” and “books” are mostly missing in 2019 for undisclosed reasons. Therefore, we drop these four product categories from our analysis at the prefecture–category level. We further reclassify the remaining 13 categories into four categories for simplicity, mostly depending on whether they were considered essential during the pandemic: (1) health-related products, including “health products,” “health foods,” and “medicine”; (2) daily essentials, including “foods” and “household products”; (3) nonessential products mostly used at home that include “sweets and alcohol,” “consumer electronics,” “furniture,” “pet products,” “hand tools and car supplies,” and “hobby supplies”; and (4) nonessential products mostly used outside the home that include “cosmetics” and “clothes.” SI Table 4 summarizes the four categories, showing the more detailed categories and some representative products in each of the four. The share of each category in total purchases is presented in Fig. 3. The shares of health products, health foods, and medicine; food and household products; and nonessential products used outside increased over time, while the share of nonessential products used at home decreased. SI Table 1 presents the summary statistics of the daily online purchases at the city level by year. The mean increased from 145 thousand yen in 2019 to 245 thousand yen in 2021.

### Covid-19 cases

The population and number of daily positive COVID-19 cases in each prefecture are taken from the Statistics Bureau of Japan [35] and the Ministry of Health, Labor and Welfare [33], respectively. We estimate the daily population in each prefecture from the yearly populations in 2015 and 2020 and the estimate for 2025, assuming a constant change rate in each five-year period. Then, we calculate the number of daily cases per 1000 persons in each prefecture. The red line in Fig. 2 indicates the one-week moving average of total daily cases in Japan. We observe five waves of COVID-19 cases during the study period: January 22-June 9, 2020; June 10-September 24, 2020; September 25, 2020-March 2, 2021; March 3-June 21, 2021; and June 22-October 30, 2021. SI Table 2 presents the summary statistics of the number of cases per 1000 at the city level.

### States of emergency

We also utilize the information taken from the Cabinet Secretariat of Japan [34] on the states of emergency declared at the prefecture level to reduce the spread of COVID-19. In Japan, a state of emergency can be declared in a prefecture by the central government after the prefectural government submits a request to the central government. After the declaration by the central government, the prefecture government can determine the details of the restrictions within the confines of the law. However, regulations during states of emergency in Japan are less strict than they are in other countries. The government can only “request” that people wear masks, stay home, work from home, and close restaurants, pubs, shops, offices, and schools but cannot force them to do so, with limited exceptions. However, because of stringent social pressure in Japan, people usually follow these requests and often stay home during a state of emergency, which was particularly true at the beginning of the pandemic. According to mobility data from mobile phones, human mobility in Tokyo declined by 50% one week after the first state of emergency was declared [36], and a survey of workers in Japan indicated that the share of workers working from home increased from 10.6% in 2017 to 35.8% in June 2020 after the pandemic began [37]. However, the decline in mobility was less significant in the later stages of the states of emergency [38].

**Figure 4:**
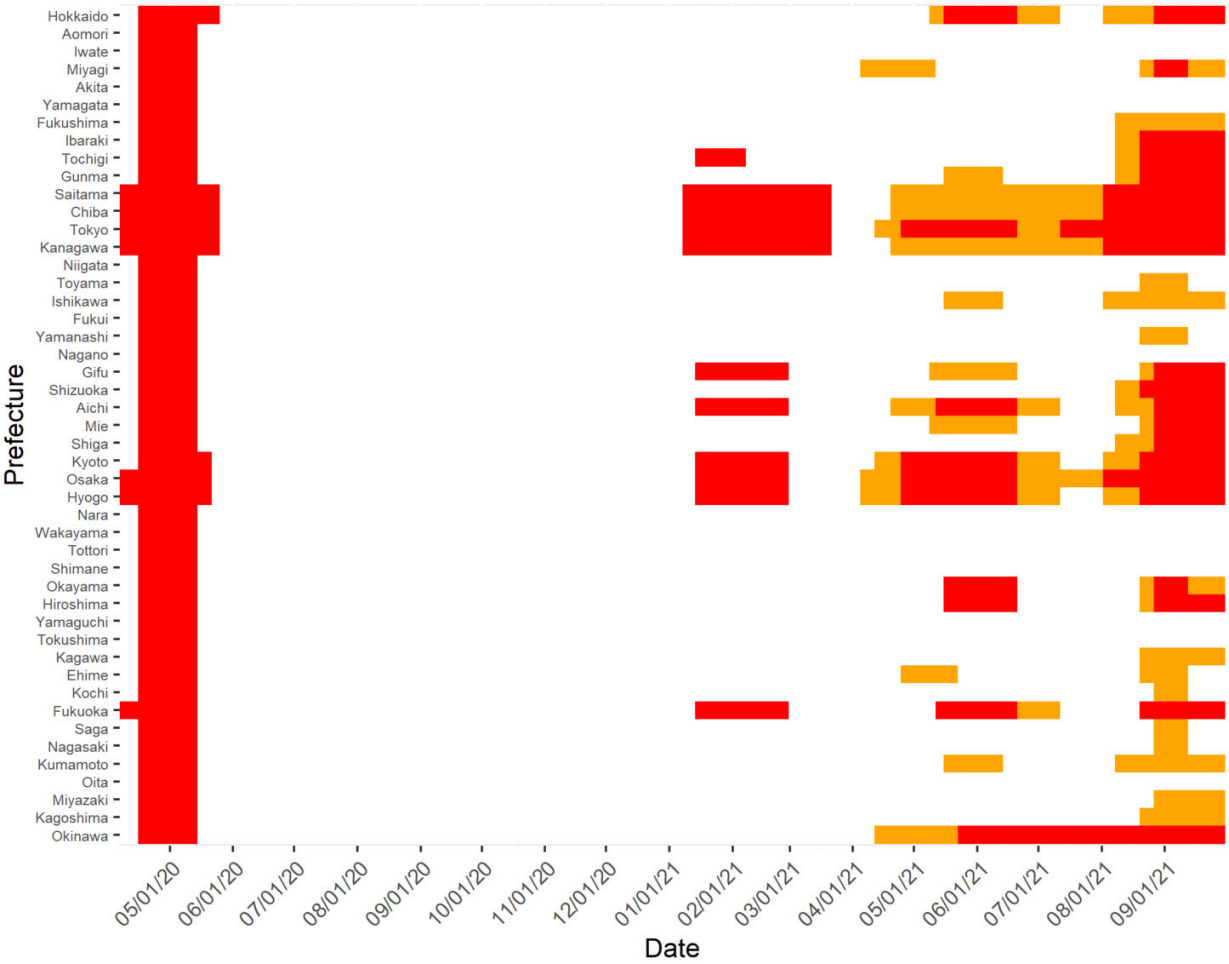
State of Emergency by Prefecture and Week. In this figure, a red cell indicates that the prefecture shown in the first column is in a state of emergency (SoE) in the week shown in the first row, running from the week of April 12, 2020, to that of October 3, 2021, whereas an orange cell indicates that it is in a semi-SoE. The weeks before April 12, 2020, and after October 3, 2021, are omitted because these periods had no SoE or semi-SoE. Data source: [34]

In response to the first wave of COVID-19 in late March 2020, an initial state of emergency was declared in seven prefectures, mostly industrial regions, including Tokyo and Osaka, on April 7, 2020. On April 16, all 47 prefectures declared a state of emergency, which was lifted in all prefectures by May 25, 2020. Then, a state of emergency was declared in some, not all, prefectures from January to March 2021, from late April to June 2021, and from July to September 2021. The shaded bars in Fig. 2 illustrate the number of prefectures in a state of emergency on each date, and the red cells in Fig. 4 indicate prefectures in a state of emergency.

In addition to states of emergency, “priority measures to prevent the spread of disease” were initiated in February 2021. These measures were less strict than were the states of emergency; thus, we call this circumstance a semistate of emergency. For example, in a semistate of emergency, restaurants and pubs are often requested not to serve alcohol after 8 pm, while in a state of emergency, they are often requested not to serve alcohol at all and to close at 8 pm (although these regulations have varied across time and prefectures). In 2021, semistates of emergency were often declared before and after a state of emergency. The orange cells in Fig. 4 show the prefectures in a semistate of emergency.

## Methods

### Simple model

Our key determinants of online purchases are two measures of pandemic shocks, i.e., the number of COVID-19 cases and the dummy (indicator) variable for a state of emergency. Conceptually, pandemic shocks affect online purchasing behavior for two major reasons, one psychological and the other physical, as summarized in the Introduction. First, in facing the pandemic, people feel anxiety and fear about future supplies and thus purchase more essential products. Second, because of government restrictions, consumers should stay home more than they did before and thus naturally rely more on online shopping. The combination of these two aspects results in an increase in online purchases, particularly of essential products. Among the two aspects, the number of cases is more related to the psychological factors of the pandemic effect because an increase in these numbers generates fear among consumers. A state of emergency is more related to the physical restrictions that affect online purchases, i.e., staying home and closing businesses. However, the two factors cannot be clearly distinguished because an increase in the number of cases may raise fear and thus lead consumers to stay home more. Additionally, a state of emergency restricts consumers physically but promotes fear among them psychologically.

The timing of the two treatments (i.e., the waves of COVID-19 cases and the stages of states of emergency) varies across time and prefectures. Therefore, to estimate the effect of the two measures on online purchases, we employ the following simple two-way fixed-effects (TWFE) estimation that incorporates individual and time fixed effects, assuming no persistent treatment effect [39]:

1 $$ \ln Y_{cpt} = \beta _{1} \ln \mathrm{CASE}_{pt} + \beta _{2} \mathrm{SoE}_{pt} + \delta _{c} + \delta _{t} + \delta _{d} + \epsilon _{cpt}, $$

where $Y_{cpt}$ is either the total purchase amount, the number of buyers, or the purchase amount per buyer in city *c* in prefecture *p* on date *t*; $\mathrm{CASE}_{pt}$ is the number of COVID-19 cases per 1000 persons in *p* on *t*; and $\mathrm{SoE}_{pt}$ is a dummy variable that takes a value of one if *p* is in a state of emergency on *t* and zero otherwise. Although the *Y* values are given at the city level, we lack data on the number of cases at the city level. However, our use of prefecture-level cases is justified because consumers are more concerned about the number of cases in their prefecture as reported every day by mass media [17], while information at the city level is rarely reported.

We take a natural log of the purchase amount, the number of buyers, and purchases per buyer, and we take a log of the number of COVID-19 cases per 1000 after adding 0.001 because, otherwise, the result may be zero. Therefore, $\beta _{1}$ can be interpreted as the quasi-elasticity of online purchases with respect to COVID-19 cases, i.e., the percentage change in the online shopping variables due to the percentage change in the number of cases. Similarly, $\beta _{2}$ is the percentage change in online shopping due to the declaration of a state of emergency. We incorporate fixed effects at the city ($\delta _{c}$), date ($\delta _{t}$), and day-of-the-week ($\delta _{d}$) levels. The city fixed effects control for any unobservable time-invariant factor, such as geographic and cultural factors, that affects online purchasing behaviors, whereas the date fixed effects represent the time trend common to all cities. The day-of-the-week fixed effects are included to capture fluctuations across days in a week.

In Equation (1), $\beta _{1}$ and $\beta _{2}$ indicate the effect of the number of COVID-19 cases and a state of emergency on the online purchases. In this simple model, we assume that once the number of COVID-19 cases becomes zero or a state of emergency is lifted, the effect on online purchases disappears; i.e., online purchases return to the trend before any COVID-19 case or state of emergency. This assumption is different from that of the standard impact evaluation literature, where the effect of a treatment persists after the treatment. We extend the model and examine the possible persistence of the effect later.

We estimate Equation (1) by ordinary least squares (OLS) regression. This estimation strategy is justified because changes in the two measures of pandemic shocks can be considered exogenous shocks to purchasing behaviors. Standard errors are clustered at the city level, incorporating possible correlations between the error terms within the city across time.

### Benchmark model

Our benchmark model used throughout this study extends Equation (1) in three ways. First, we examine heterogeneity in the effect of the COVID-19 cases and states of emergency across time, following the recent literature on impact evaluation [39–41]. Because the pandemic lasted for one and a half years in the sample period, consumers may have coped with the pandemic and thus may have responded less to the pandemic shocks as time progressed [24]. In the presence of heterogeneity in treatment effects across time, the coefficient of the treatment variables in Equation (1) is the weighted average of the time-varying effects and can be biased [39, 40, 42]. Therefore, we incorporate time heterogeneity in the effect of pandemic shocks by distinguishing among the number of COVID-19 cases across the five waves and among the states of emergency in different months.

Second, we include dummy variables for before and after a state of emergency. Specifically, we incorporate a dummy variable that takes a value of one if prefecture *p* declared a state of emergency on date $t+1$ (i.e., *p* is one week before a state of emergency is declared) and zero otherwise. Similarly, three other dummy variables for 2-4 weeks before a state of emergency are included. We include pre-state-of-emergency dummies to check whether a state of emergency has any effect prior to its declaration through the anticipation effect. Usually, a few days before the central government declares a state of emergency in a prefecture, its governor requests a declaration from the central government in response to increasing cases in the prefecture. Therefore, a state of emergency is generally predictable a few days before its declaration. In addition, we incorporate dummy variables for 1-4 weeks after any state of emergency. Doing so enables us to test whether the possible effect of a state of emergency persisted even after the declaration was lifted, a major hypothesis in this study, as described in the Introduction. The absence of the post-state of emergency effect is a major assumption of static TWFE estimations using equations such as (1) and thus should be tested.

Finally, we examine spillover effects, i.e., whether consumer behavior in prefecture *p* is affected by any COVID-19 case or state of emergency in neighboring prefectures because of fear of the spread of COVID-19 and the resulting state of emergency in *p* in the future. If spillover effects exist, then $\beta _{1}$ and $\beta _{2}$ in Equation (1) may be biased [43]. For this purpose, we include in Equation (1) the total number of cases in prefectures that share any border with prefecture *p* and the number of neighboring prefectures in a state of emergency. As in the previously defined variables, we distinguish among the number of cases in neighboring prefectures by wave and among the number of neighboring prefectures in a state of emergency by month.

Accordingly, our city-level analysis is based on the following equation:

2 $$ \begin{aligned}[b] \ln Y_{cpt} ={} &\sum _{w} \beta _{1w} \ln \mathrm{CASE}_{ptw} + \sum_{m} \beta _{2m} \mathrm{SoE}_{ptm} \\ &{}+ \sum_{y=2020}^{2021} \sum _{k=1}^{4} \lambda _{1yk} \mathrm{PreSoE}_{ptyk} + \sum_{y=2020}^{2021} \sum_{k=1}^{4} \lambda _{2yk} \mathrm{PostSoE}_{ptyk} \\ &{}+ \sum_{w} \mu _{w} \ln \biggl( \sum_{q \in Q_{p}} \mathrm{CASE}_{qtw} \biggr) + \sum_{m} \mu _{m} \sum _{q \in Q_{p}} \mathrm{SoE}_{qtm} + \delta _{c} + \delta _{t} + \delta _{d} + \epsilon _{cpt}, \end{aligned} $$

where $\mathrm{CASE}_{ptw}$ is the number of COVID-19 cases per 1000 persons in prefecture *p* on date *t* in wave *w* (*w* is from one to five), and $\mathrm{SoE}_{ptm}$ is the dummy variable that takes a value of one if *p* is in a state of emergency in *t* in month *m*. $\mathrm{PreSoE}_{ptyk}$ and $\mathrm{PostSoE}_{ptyk}$ are one if prefecture *p* is *k* weeks before and after a state of emergency, respectively, in *t* in year *y* and zero otherwise. For example, $\mathrm{PreSoE}_{pt,2020,2}$ is one if prefecture *p* is two weeks before a state of emergency on date *t* in 2020. Accordingly, coefficient $\lambda _{1,2020,2}$ indicates the effect of a state of emergency in 2020 on the online purchases two weeks after the state of emergency. $Q_{p}$ is the set of prefectures neighboring *p*. Therefore, $\sum_{q \in Q_{p}} \mathrm{CASE}_{qtw}$ represents the sum of the COVID-19 cases in neighboring prefectures; thus, its coefficient, $\mu _{w}$, indicates the spillover effect from neighboring prefectures in wave *w*. Semistates of emergency, which are less restrictive than are states of emergency (refer to the previous section), are also included in the analysis but are not distinguished by declaration month for simplicity.

### Econometric issues

Several econometric issues remain in estimating Equation (2). First, from April 16 to May 14, 2020, all 47 prefectures in Japan were in a state of emergency. Because we cannot compare prefectures with and without a state of emergency during this period, we drop this time period from the sample [39].

Second, when the timing of the treatments varies, the estimate of the treatment variables without any consideration of the time variation may be biased. We alleviate this problem by incorporating the time-variant effects of the COVID-19 shocks, as explained earlier. Moreover, bias may be minimal in this study because the prefectures that declared a state of emergency earlier than others lifted it later than did the others in all three stages(Fig. 4). Therefore, our estimation avoids the biases arising from a comparison among the prefectures that were in a state of emergency but are currently not and the prefectures that are currently in a state of emergency, assuming that the former would not have been treated [42].

Finally, an important identifying assumption of our estimation is a parallel trend prior to the treatment. If any difference arises in the prepandemic trend between the prefectures that were severely hit by COVID-19 and thus declared a state of emergency for a long time and the other prefectures, *β*s in Equation (2) may not simply be the effect of the pandemic shocks but may include the systematic difference between the two prefecture types [41]. We test this possibility using the data prior to the pandemic, i.e., from January to December 2019. Specifically, we regress the purchase amount, the number of buyers, and the purchase amount per buyer on the dummy variable for the last half of 2019; the dummy variable for the prefectures that declared the state of emergency for more days than the median prefecture (47 days); and the interaction term between the two dummies. SI Table 5 shows that the interaction term is not significantly different from zero in any of the regressions. Therefore, the pretreatment parallel-trend assumption is satisfied.

### Heterogeneity across products

The analysis at the prefecture–product level estimates an equation similar to Equation (2) but assumes the heterogeneous effects of the COVID-19 cases and states of emergency on the different products, using the four product categories broadly defined in the previous section: health-related products, daily essentials, nonessential products used mostly at home, and nonessential products used mostly outside the home (SI Table 4). Conceptually, however, because of increasing needs for essential products, particularly health products and health foods, the effect on these products is predicted to be greater than that on the nonessential products. To test this hypothesis, all the independent variables except for the fixed effects are interacted with the dummy variables for the product categories. Product fixed effects are also included, and city fixed effects are replaced with prefecture fixed effects. Standard errors are clustered at the prefecture and date levels, controlling for a correlation between the error terms across products. Specifically, the estimation equation for the prefecture–product level estimates is given by the following:

3 $$\begin{aligned} \ln Y_{pjt} =&\sum _{j} \sum_{w} \beta _{1jw} \ln \mathrm{CASE}_{ptw} \times D_{j} + \sum_{j} \sum_{m} \beta _{2m} \mathrm{SoE}_{ptm} \times D_{j} \\ &{}+ \sum_{j} \sum _{y=2020}^{2021} \sum_{k=1}^{4} \lambda _{1jyk} \mathrm{PreSoE}_{ptyk} \times D_{j} + \sum_{j} \sum _{y=2020}^{2021} \sum_{k=1}^{4} \lambda _{2jyk} \mathrm{PostSoE}_{ptyk} \times D_{j} \\ &{}+ \sum_{j} \sum _{w} \mu _{j}w \ln \biggl( \sum _{q \in Q_{p}} \mathrm{CASE}_{qtw} \biggr) \times D_{j} + \sum_{j} \sum _{m} \mu _{j}m \sum _{q \in Q_{p}} \mathrm{SoE}_{qtm} \times D_{j} \\ &{}+ \delta _{p} + \delta _{j} + \delta _{t} + \delta _{d} + \epsilon _{pjt}, \end{aligned}$$

where *j* represents product categories and $D_{j}$ is the dummy variable for product *j*.

## Results

### City-level analysis

**Figure 5:**
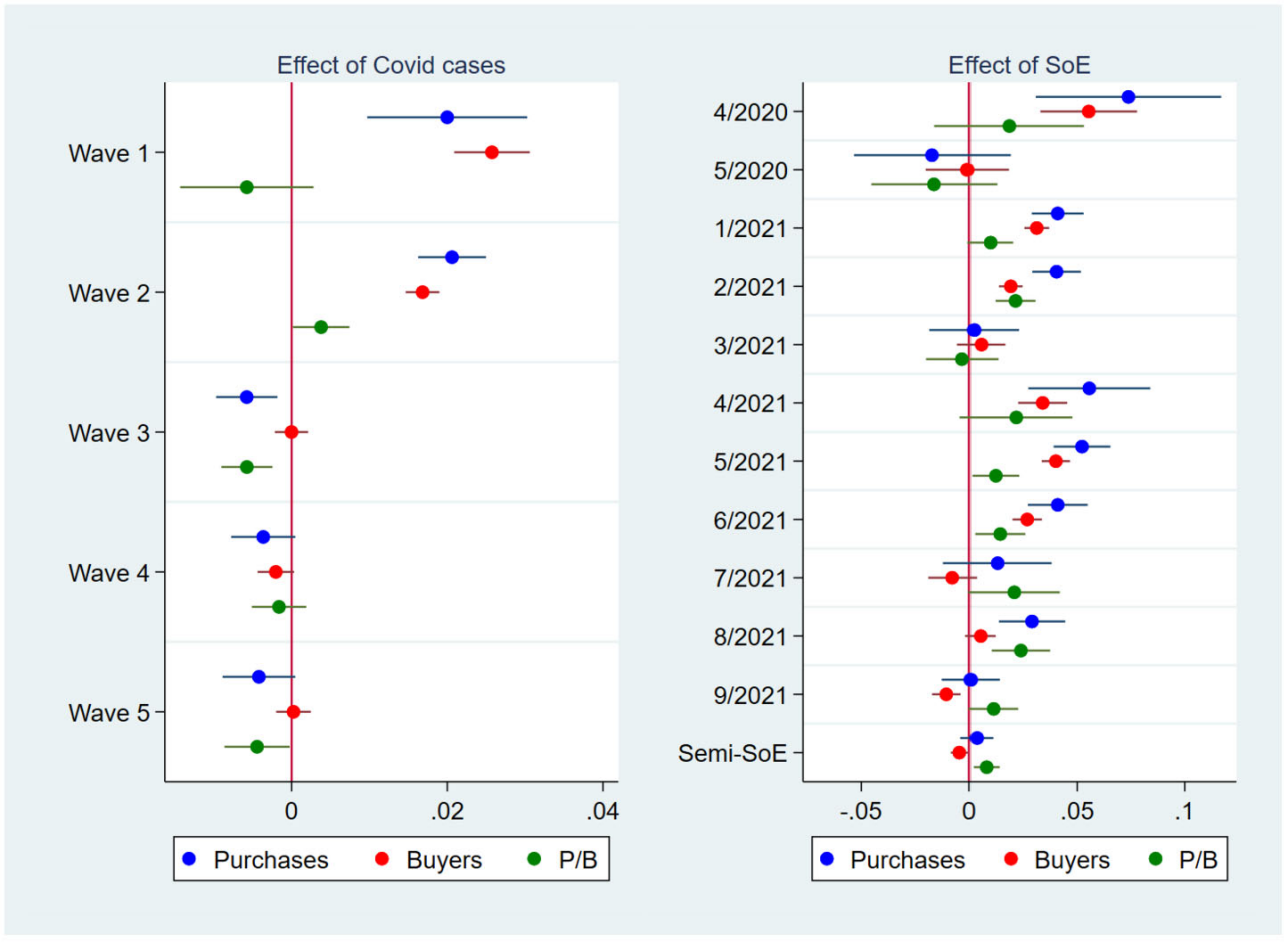
Effects of the Number of COVID-19 Cases and the Declaration of a State of Emergency on the Purchase Amount, the Number of Buyers, and the Purchase Amount per Buyer. This figure illustrates the results from the regressions of the purchase amount, the number of buyers, and the purchase amount per buyer in logs at the city-date level. The left panel shows the point estimates and 95% confidence intervals of the effect of the number of COVID-19 cases per 1000 persons averaged over one week on the purchase amount (blue), the number of buyers (red), and the purchase amount per buyer (green) in the five waves of COVID-19 in the sample period. The right panel shows the coefficients and 95% confidence intervals of the declaration of a state of emergency (SoE), separated into the months of the sample period and a semi-SoE, which is less restrictive than an SoE. The number of COVID-19 cases and the declaration of an SoE are given at the prefecture–date level. We take a log of the number of COVID-19 cases per 1000 persons plus 0.001 to incorporate possible nonlinear relationships. In all the regressions, the other independent variables are the number of neighboring prefectures in an SoE; the dummy variables for 1-4 weeks before and after the declaration of an SoE and a semi-SoE; and the city, date, and day-of-the-week fixed effects. The standard errors are clustered at the city level. N = 1,091,171

**Figure 6:**
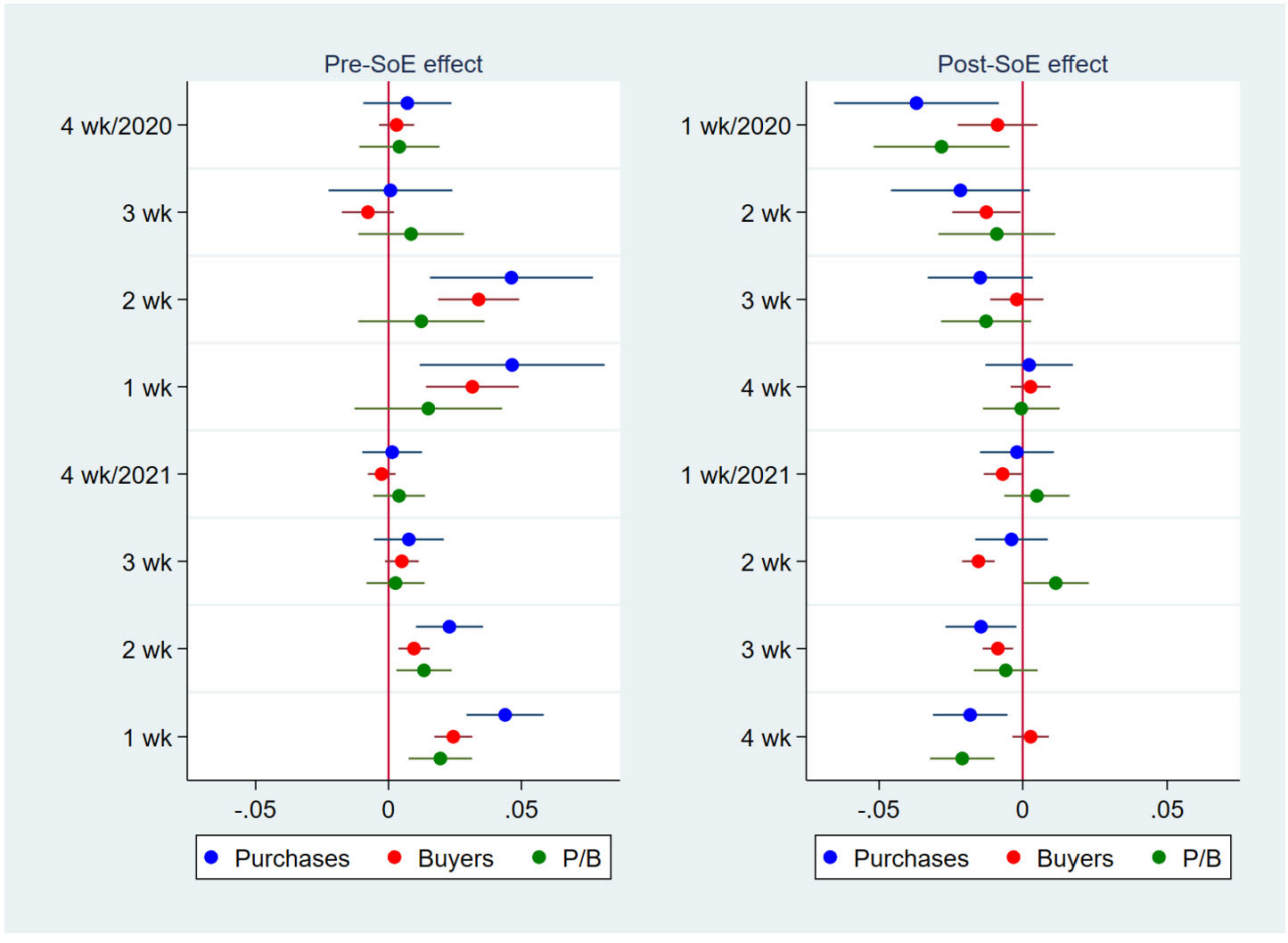
Pre- and Posttreatment Effects of the Declaration of a State of Emergency on the Purchase Amount, the Number of Buyers, and the Purchase Amount per Buyer. This figure illustrates the results from the regressions of the purchase amount, the number of buyers, and the purchase amount per buyer in logs at the city–date level. The left panel shows the point estimates and 95% confidence intervals of the dummies for 1-4 weeks before the declaration of a state of emergency (SoE) on the purchase amount (blue), the number of buyers (red), and the purchase amount per buyer (green). The right panel shows those of the dummies for 1-4 weeks after the end of an SoE. The dummies for before and after an SoE are given at the prefecture–date level. For example, “4 wk/2020” on the x-axis of the left panel means 4 weeks before an SoE in 2020, whereas “1wk/2020” in the right panel means 1 week after an SoE in 2020. In all the regressions, the other independent variables are the number of COVID-19 cases per 1000 persons; the dummies for an SoE; the number of neighboring prefectures in an SoE; and the city, date, and day-of-the-week fixed effects. The standard errors are clustered at the city level. N = 1,091,171

We first run the TWFE estimation of Equation (2) at the city level and illustrate the results in Figs. 5, 6, and 8. The detailed numerical results are presented in SI Table 6.

First, the blue, red, and green dots in the left panel of Fig. 5 indicate the point estimates of the effect of each of the five waves of COVID-19 cases on the purchase amount, the number of buyers, and the purchase amount per buyer in log, respectively, while the lines show the corresponding 95% confidence intervals. The results reveal a positive and significant effect of the number of COVID-19 cases on online purchases in Waves 1 (January-June, 2020) and 2 (June-September, 2020). In stark contrast, the effect of the COVID-19 cases in Waves 3 (September 202-March 2021), 4 (March-June 2021), and 5 (June-October 2021) was either nonsignificant or significant but slightly negative. Thus, the number of cases did not increase online purchases one year after the beginning of the pandemic.

The point estimates of the effect of the COVID-19 cases in Waves 1 and 2 were 0.020 and 0.021, respectively, implying that a doubling of the number of COVID-19 cases per person increased the online purchases by approximately two percent. To highlight more clearly the size of these effects, we take the example of Shinjuku Ward in Tokyo, a hotspot of COVID-19 cases. At the beginning of Wave 2, June 10, 2020, the number of cases per 1000 in the ward was 0.0014, and this figure increased to a peak of 0.0264 on August 5. These data mean that the log of the number of cases plus 0.001 increased by 2.44 ($=\log 0.0274 - \log 0.0024$) from June 10 to August 5, 2020. In the same period, online purchases in logs in this ward increased by 1.09. Considering the estimated coefficient of the log of the number of cases, 0.021, these figures imply that 4.7% ($=0.021 \times 2.44/1.09$) of the increase in online purchases was due to the increase in the COVID-19 cases. Using the average figures across cities, we obtain a similar quantitative result. Therefore, although the effect of the COVID-19 cases in Waves 1 and 2 was significant, its size may not necessarily be large.

The effect on the total purchase amount can be decomposed into the effect on the number of buyers and on the purchase amount per buyer. Comparing the blue dots and lines (i.e., the effect on total purchases) with the red and green dots and lines (the number of buyers and purchases per buyer, respectively), we find that the positive effect of the number of cases in the first two waves on purchases is attributable to its positive effect on the number of buyers, not on the purchases per buyer. However, the number of buyers defined here is the number of buyers in a day. Therefore, whether the positive effect on the number of buyers reflects new online shopping platform users or more frequent usage by existing users remains unclear.

The right panel of Fig. 5 presents the effect of the states of emergency by month. Looking at the blue dots and lines, we find that the first state of emergency declared in April 2020 raised the purchase amount on the online shopping platform by 7.4%, while its effect became nonsignificant one month later, in May. Similarly, the second-stage state of emergency had a positive effect on the purchase amount in the early period in January and February 2021 but lost its positive effect later in March. The effect of the third-stage state of emergency that started in late April 2021 was also positive for the first three months but became nonsignificant or nearly zero later. The red and green dots and lines in the panel suggest that the positive effect of a state of emergency in its earlier period on the purchase amount mostly stems from its effect on the number of buyers, not on the purchases per buyer. A semistate of emergency did not affect online purchases (right panel, bottom), possibly because of its less strict regulations.

Comparing the effects of the number of cases and states of emergency, we find that although Waves 3-5 of COVID-19 cases in 2021 had no positive effect on online purchases, the states of emergency in 2021 did, particularly in the early period of each stage. One possible reason for this contrast is that an increase in the number of cases no longer encouraged consumers to stay home one year into the pandemic because they learned to cope with their pandemic-related fears, while states of emergency continued to prompt consumers to stay home and increased the necessity of using online shopping.

Figure 6 presents the effect of the pre- and post-states of emergency periods. The left panel shows that the purchase amount, the number of buyers, and the purchase amount per buyer in the prefectures in a state of emergency were not systematically different from those in the other prefectures three and four weeks before the declaration of a state of emergency in either 2020 or 2021. In contrast, one or two weeks before such a declaration, the prefectures that then declared a state of emergency sometimes exhibited higher total purchases, a higher number of buyers, or more purchases per buyer than did other prefectures, possibly because people predicted the future declaration of a state of emergency and thus changed their behaviors one to two weeks prior to the declaration. This result is consistent with the finding from the mobile phone data that human mobility started to decline a few weeks before the first state of emergency was declared [44, 45].

The right panel of Fig. 6 shows that the contemporaneous positive effect of a state of emergency on the purchase amount and the number of buyers found in the right panel of Fig. 5 became nonsignificant or even significantly negative after the state of emergency was lifted. The effect of a state of emergency on online purchasing behavior was temporary and did not persist after its lifting (or after a wave of COVID-19 cases because a state of emergency is usually lifted when the number of cases declines substantially).

**Figure 7:**
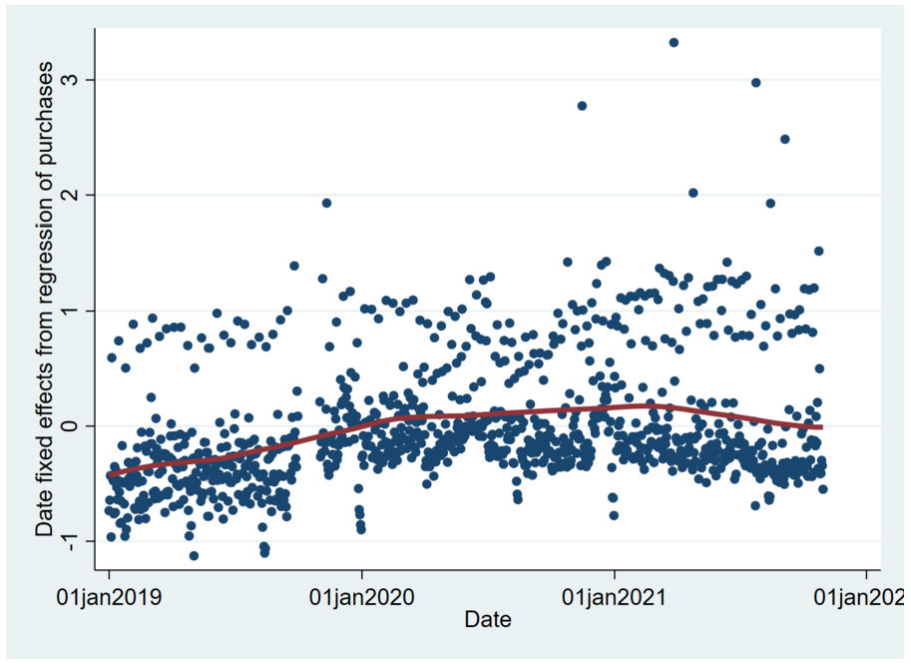
Date Fixed Effects from the Regression of the Purchase Amount. In this figure, the blue dots represent the date fixed effects estimated from the regressions of the purchase amount at the city level for each date, and the red line represents the fitted relationship between date fixed effects and the dates using a nonparametric local–linear kernel regression. The independent variables are the number of COVID-19 cases per 1000 persons, the declaration of a state of emergency (SoE); the number of neighboring prefectures in an SoE; the dummy variables for 1-4 weeks before and after the declaration of an SoE and a semi-SoE; and the city, date, and day-of-the-week fixed effects. The standard errors are clustered at the city level. N = 1,091,171

However, this result is not sufficient to conclude that the effect of the pandemic on online purchasing behavior did not persist because the overall trend of the online purchases could have increased after the pandemic despite this result. To test this possibility, in Fig. 7, we plot the relationships among the date fixed effects from the estimation of Equation (2), $\delta _{t}$, and date *t* and a fitted line using a nonparametric local–linear kernel regression. The figure shows that the date fixed effects, or the sum of any time-variant factor that determines that the online purchases excluding the effect of the number of COVID-19 cases and states of emergency, were stable after the COVID-19 pandemic started in March 2020. A parametric estimation also cannot reject the hypothesis that the average of the date fixed effects in the pre- and postpandemic periods are the same. These results suggest that the time trend in online purchases did not change because of the COVID-19 pandemic, confirming its temporary effect on online purchasing behavior.

**Figure 8:**
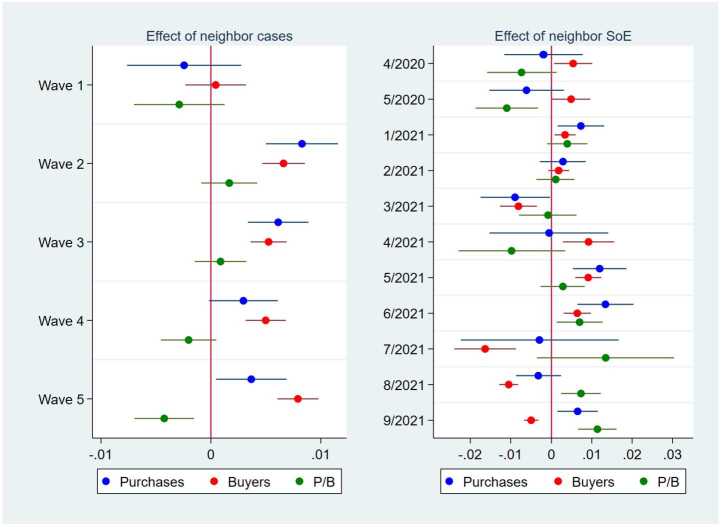
Effects of the Declaration of a State of Emergency in Neighboring Prefectures on the Purchase Amount, the Number of Buyers, and the Purchase Amount per Buyer. This figure illustrates the point estimates and 95% confidence intervals of the effect of the number of prefectures that were in a state of emergency (SoE) and shared any border with the focal prefecture in different months on the purchase amount (blue), the number of unique buyers (red), and the purchase amount per unique buyer (green) in logs. In all the regressions, the other independent variables are the dummies for an SoE; the dummies for before an SoE; the number of COVID-19 cases per 1000 persons; the number of neighboring prefectures in an SoE; and the prefecture, product, date, and day-of-the-week fixed effects. The standard errors are clustered at the city level. N = 1,091,171

Finally, a state of emergency in neighboring prefectures did not affect the online purchases in 2020, although it had a positive effect in January, February, May, June, and September 2021 (Fig. 8). However, unlike the effect of a state of emergency in consumers’ own prefectures, the effect of neighboring prefectures’ states of emergency did not show a clearly declining trend over time. The effect of a state of emergency in a prefecture clearly still spilled over to its neighbors, possibly because consumers in the neighboring prefectures begin preparing for a future state of emergency and thus increased their use of online shopping. Therefore, the spillover effect must be controlled for to eliminate bias when estimating the effect of COVID-19 shocks.

To check the robustness of these results, we use the two treatment variables separately; i.e., we used each of the two in one regression rather than using both together in one regression. The results from these alternative specifications are presented in SI Figs. 1 and 2, which correspond to Figs. 5 and 6, respectively. These results are essentially the same as those from the benchmark specifications, confirming our main conclusions.

### Prefecture–product-level analysis

**Figure 9:**
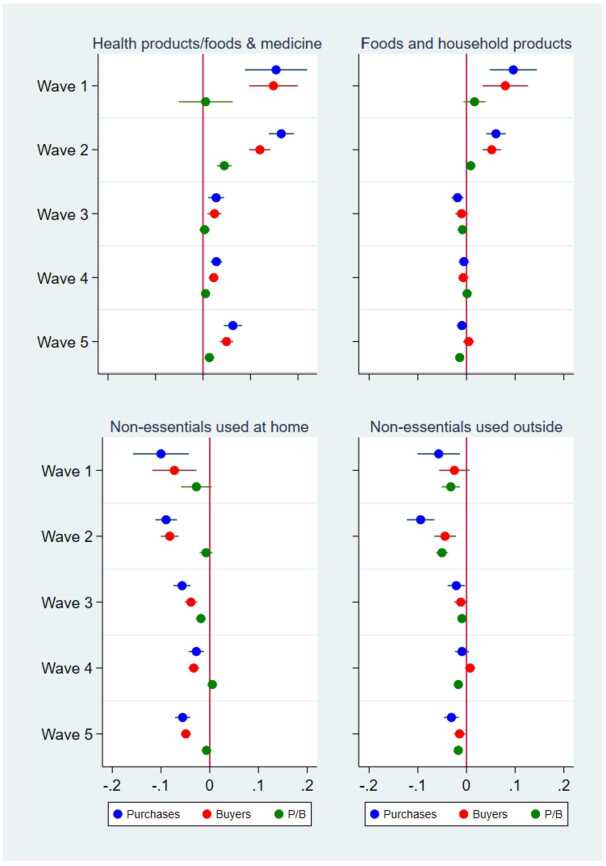
Effects of the Number of COVID-19 Cases on the Purchase Amount, the Number of Buyers, and the Purchase Amount per Buyer by Product Category. This figure illustrates the results from the regressions of the purchase amount, the number of buyers, and the purchase amount per buyer in logs at the prefecture–product–date level by the product category. The dots and lines indicate the coefficients and 95% confidence intervals of the effect of the dummies for 1-4 weeks after a state of emergency (SoE) in different months on the purchase amount (blue), the number of buyers (red), and the purchase amount per buyer (green). In all the regressions, the other independent variables are the dummies for an SoE; the dummies for before an SoE; the number of COVID-19 cases per 1000 persons; the number of neighboring prefectures in an SoE; and the prefecture, product, date, and day-of-the-week fixed effects. The standard errors are clustered at the prefecture and date levels. N = 188,437

We now turn to the prefecture–product-level analysis to examine heterogeneity in the effect of the COVID-19 shocks across products and estimate Equation (3). Figure 9 shows the effect of the number of COVID-19 cases for each of the four product categories. The upper two panels indicate the results for essential products, i.e., (1) health products, health foods, and medicine and (2) food and household products, whereas the lower two panels are the results for nonessential products. The blue dots and lines clearly indicate that the COVID-19 cases had a large positive effect on the purchasing of essential products, while the effect on the purchasing of nonessential products was mostly negative. These results suggest that consumers with pandemic-related fears purchased more essential products online but limited their purchases of nonessential products. The point estimates imply that a doubling of the number of cases resulted in an increase in online purchases of essential products by more than 10% in the first wave. However, the positive effect on essential products declined to nonsignificant or quite low levels after the third wave. As in the previous analysis, the positive effect on total purchases was mostly attributable to the effect on the number of buyers (red) rather than on the purchases per buyer (green).

**Figure 10:**
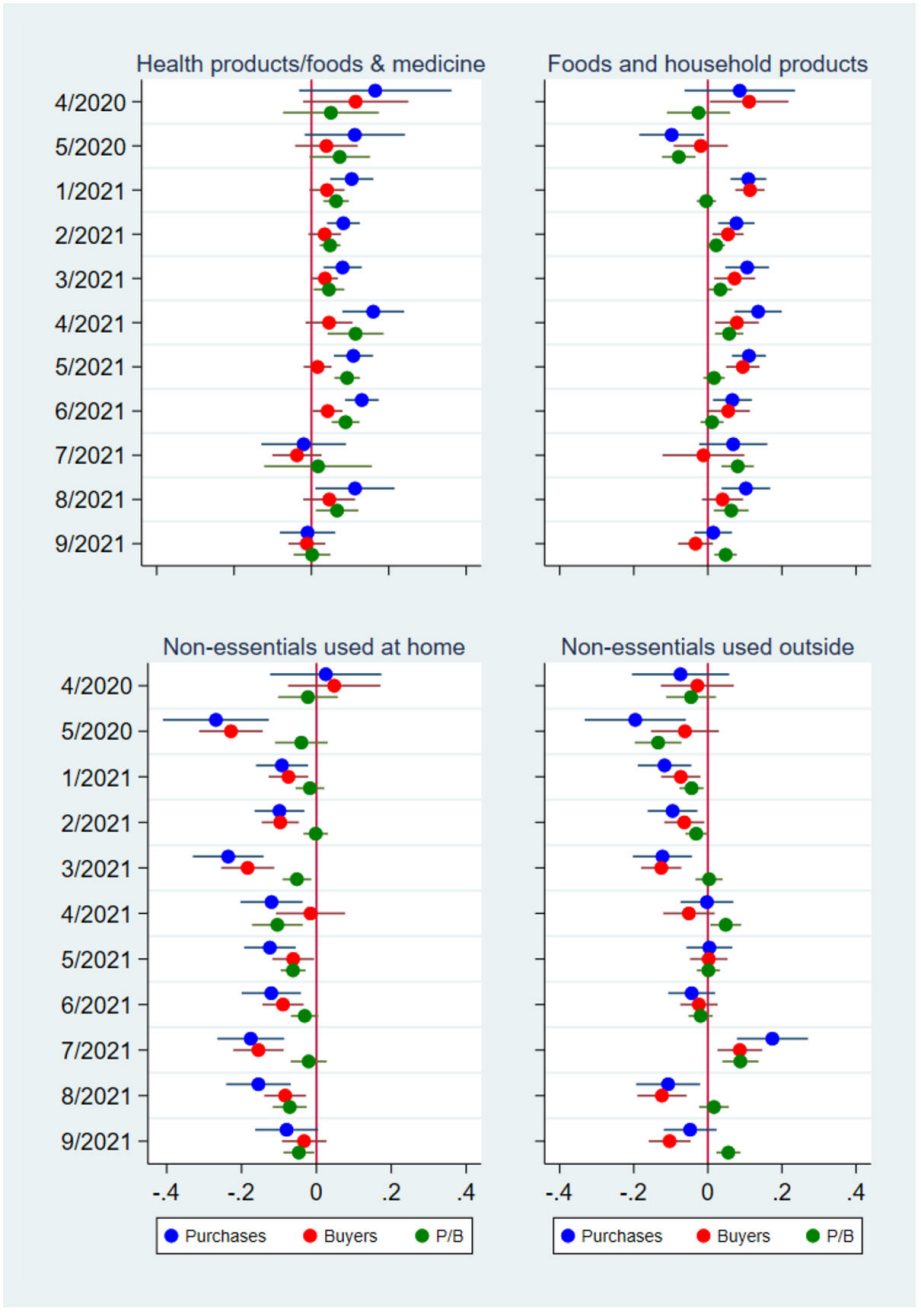
Effects of a State of Emergency on the Purchase Amount, the Number of Buyers, and the Purchase Amount per Buyer by Product Category. This figure illustrates the results from the regressions of the purchase amount, the number of buyers, and the purchase amount per buyer in logs at the prefecture–product–date level by the product category. The dots and lines indicate the coefficients and 95% confidence intervals of the effect of the dummies for 1-4 weeks after a state of emergency (SoE) in different months on the purchase amount (blue), the number of buyers (red), and the purchase amount per buyer (green). In all the regressions, the other independent variables are the dummies for an SoE; the dummies for before an SoE; the number of COVID-19 cases per 1000 persons; the number of neighboring prefectures in an SoE; and the prefecture, product, date, and day-of-the-week fixed effects. The standard errors are clustered at the prefecture and date levels. N = 188,437

Figure 10 also shows that the effect of states of emergency on online purchases (represented by the blue dots) was positive for essential products, except in 2020, but was mostly negative for nonessential products. Moreover, the positive effect on essential products was more persistent over time than was the overall effect of the states of emergency on total purchases (Fig. 5, right panel) and the effect of the COVID-19 cases on the purchases of essential products (Fig. 9). However, the positive effect eventually disappeared in September 2021.

**Figure 11:**
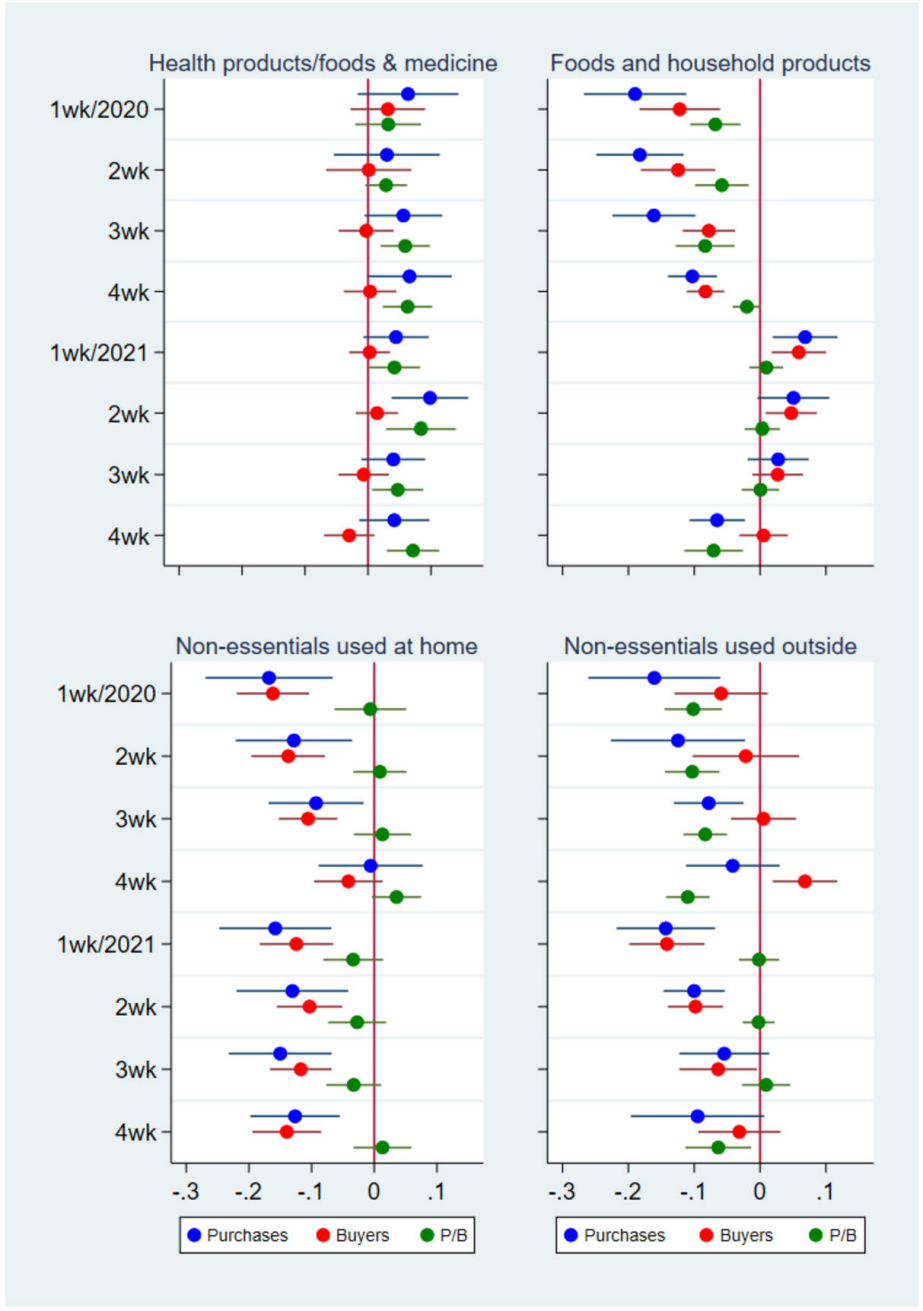
Posttreatment Effects of a State of Emergency on the Purchase Amount Purchases, the Number of Buyers, and the Purchase Amount per Buyer by Product Category. This figure illustrates the results from the regressions of the purchase amount, the number of buyers, and the purchase amount per buyer in logs at the prefecture–product–date level by the product category. The dots and lines indicate the coefficients and 95% confidence intervals of the effect of the dummies for 1-4 weeks after a state of emergency (SoE) in different months on the purchase amount (blue), the number of buyers (red), and the purchase amount per buyer (green). “1wk/2020” on the x-axis means 1 week after an SoE in 2020. In all the regressions, the other independent variables are the dummies for an SoE; the dummies for before an SoE; the number of COVID-19 cases per 1000 persons; the number of neighboring prefectures in an SoE; and the prefecture, product, date, and day-of-the-week fixed effects. The standard errors are clustered at the prefecture and date levels. N = 188,437

Finally, Fig. 11 shows the effect of the post-state of emergency. The effect of a state of emergency on the purchases of health products/foods and medicine 1-4 weeks after it was lifted was positive and mostly nonsignificant at the 5% level. Although the effect was sometimes significant at the 10% level, we used a significance level of 5%, not 10%, because of the large number of observations in our estimations. Even if we used the 10% significance level and concluded that the online purchases of health-related products persistently increased with each state of emergency, this conclusion would not be inconsistent with our previous conclusion that the effect of states of emergency on total online purchases was not persistent. The reasoning is that the purchases of other categories of products either did not change or even declined in the post-state-of-emergency period and because the share of health-related products of total online purchases was not large (Fig. 3). The weakly persistent increase in the purchases of health-related products was canceled out by the decrease in the purchases of other products; hence, the effect on total purchases was not significant in the post-state of emergency period (Fig. 6, right panel).

## Discussion

The results in the previous section suggest that the COVID-19 pandemic stimulated purchases on a major online shopping platform in Japan (Fig. 5). This result is most likely because consumers facing increasing numbers of positive COVID-19 cases and declarations of a state of emergency stayed home more and relied more on online shopping than did other consumers. Our further analysis clarifies that the increase in daily online purchases was attributed to an increase in the number of buyers on the platform purchasing essential products, such as health products, food, and household products (Figs. 9 and 10). In contrast, the average purchase amount per buyer did not increase, and the purchase amount of nonessential products often declined during the pandemic. Our findings are quite consistent with those of earlier studies on the pandemic effect on consumption in general [1, 3, 6–14] and on online consumption [5, 20–23].

Moreover, their positive effect on online purchases was not persistent but transitory, decreasing as consumers became accustomed to the pandemic and disappearing when a wave of the pandemic subsided and states of emergency were lifted (Fig. 6). When we examined the effect by product category, we observed heterogeneity in the pandemic effect and weak evidence of a persistent increase in the online purchases of health-related products after a state of emergency (Fig. 11). However, because the number of nonessential product purchases generally declined after a state of emergency, the total, long-term effect was zero. In addition, the overall time trend in online shopping utilization did not increase after the pandemic once the temporary effects of the COVID-19 shocks were eliminated (Fig. 7).

These results suggest that the pandemic has not permanently changed online purchasing behavior in Japan. Consumers purchase essential products online more often when they are psychologically and physically constrained; i.e., they stayed home because they fear infection and are under states of emergency. However, consumers returned to offline shopping once the psychological and physical restrictions were relaxed.

Our conclusion is in line with that of an earlier study on Japan by Watanabe et al. using credit card transaction data [29]. However, our analysis differs from theirs in that we use higher-frequency data (daily) for a longer period (January 2019 to October 2021) than theirs, with eight time points from January 2019 to October 2020. Accordingly, our data period covers several waves of COVID-19 and several stages of states of emergency in 2021 (Fig. 2) that are not covered by Watanabe et al. As a result, we explicitly incorporate the number of COVID-19 cases and states of emergency as determinants of online purchases, finding their diminishing effect over time, and we further test whether the effect of the shocks persist after the shocks were temporarily removed. The analysis of Watanabe et al. was based on transition probabilities of purchasing behavior between several time points and does not test any of these effects statistically. In addition, none of the studies carried out in other countries has examined the effect of the number of cases, states of emergency, or post-states of emergency, so no regional variations of these variables have been considered [20–24].

Two other possible reasons may explain the temporary effect of the pandemic shocks that can be generalized to other shocks and other countries. First, online shopping is quite convenient once users pay initial costs, i.e., setting up accounts and learning how to use the platform. However, compared to offline shopping, online shopping has several disadvantages, such as delayed possession, a lack of social interactions, privacy and security risks, and the difficulty of processing online information [25–27, 29]. Therefore, consumers who are forced to shop online during periods of strong restrictions may return to offline shopping when the restrictions are lifted if the variable costs of online shopping are sufficiently high for them. These costs are presumably high in Japan. A survey by the Ministry of Economy, Trade and Industry in 2020 after the COVID-19 pandemic found that 88% of the respondents were afraid that their private information was exposed through online shopping, a proportion that is not very different from that found in 2017 [46].

Second, facing a large shock such as the COVID-19 pandemic, people usually react strongly at first, but their reaction weakens after they learn how to cope with the situation [6, 24, 28]. Our results clearly show that consumers adapted to the pandemic because we find that the effect of the case number and states of emergency diminished over time and ultimately disappeared (Fig. 6). Initially, the pandemic generated anxiety and fear, which drove panic buying and hoarding via online shopping channels [3, 10, 15–17], but consumers became accustomed to the pandemic and thus began to reduce such reactions. This presumption is supported by the data on human mobility in Japan. Although human mobility declined drastically in the first stage of the first state of emergency [36, 45], the decline was limited after the second stage [38, 44]. These findings reveal that as consumers experienced several waves of the pandemic and several stages of emergency, they stayed home less and shopped in person more. Therefore, in the long run, consumers are expected to return to the same offline shopping habits that they had before the pandemic.

From the perspective of consumer welfare, these two reasons provide different implications. The first reason, i.e., the disadvantages of online shopping, implies that consumers’ decisions are rational and that consumers’ welfare is maximized by their temporal response to the pandemic. However, the second reason, i.e., the overvaluation of the pandemic shock, implies that consumers are irrational; thus, alleviating the overreaction could improve their welfare.

In addition, one managerial implication of this study for online shopping platforms in general is that online sales may decline after the COVID-19 pandemic. To make the temporary increase in online sales during the pandemic more persistent, more useful tools, such as one-day deliveries and online social communities, should be employed to lower the current costs of online shopping. In addition, using more innovative technologies, such as virtual reality (VR), 3-dimensional (3D) images, and the metaverse, can provide consumers with information that can be processed more easily without high levels of cognitive skills [25]. Finally, risks to privacy and security in online shopping are another key concern for consumers and thus should be lowered.

## Supplementary Information

Below is the link to the electronic supplementary material.

Supplementary information (PDF 429 kB)

## Data Availability

The data supporting the findings of this study are available from Yahoo! Japan, but restrictions apply to their availability, as they were used under license for the current study and are not publicly available. The data are, however, available from the authors upon reasonable request and with permission from Yahoo! Japan.
