# Supplementary material for: Has Covid-19 permanently changed online purchasing behavior?
Source: EPJ Data Sci. 2023 Jan 16;12(1):1. doi: 10.1140/epjds/s13688-022-00375-1 (PMC9841963; doi:10.1140/epjds/s13688-022-00375-1)
Supplement: Supplementary file 1 — Supplementary information (PDF 429 kB) [file 13688_2022_375_MOESM1_ESM.pdf]

Supplementary Information

Has COVID-19 Permanently Changed Online Purchasing Behavior?

SI Table 1: Summary Statistics: Online Purchases at the City Level[1]

	N	Mean	SD	Min	Max
2019	368060	145,423.2	434,547.4	600	46,812,600
2020	380830	206,259.8	690,380.2	800	116,819,900
2021	342281	244,789.0	1,181,328.8	400	200,115,900
Total	1091171	197,825.1	818,192.1	400	200,115,900

SI Table 2: Summary Statistics: Number of Positive Cases of COVID-19 per 1,000 people at the City Level[2]

	N	Mean	SD	Min	Max
2019	368060	0.00000	0.00000	0	0
2020	380830	0.00395	0.00775	0	.0679338
2021	342281	0.03152	0.04866	0	.4702274
Total	1091171	0.01127	0.03088	0	.4702274

SI Table 3: Summary Statistics: Number of Positive Cases of COVID-19 per 1,000 people in log at the City Level[3]

	N	Mean	SD	Min	Max
2019	368060	-6.908	0.000	-6.907755	-6.907755
2020	380830	-5.995	1.046	-6.907755	-2.674608
2021	342281	-4.157	1.223	-6.907755	-.7524145
Total	1091171	-5.726	1.456	-6.907755	-.7524145

[1]This table shows the number of observations (N), mean, standard deviation (SD), minimum (Min) and maximum (Max) of the purchase amount at the city level by year and in total yen.

[2]This table shows the number of observations (N), mean, standard deviation (SD), minimum (Min) and maximum (Max) of the number of positive cases of COVID-19 per 1,000 people at the city level by year and in total.

[3]This table shows the number of observations (N), mean, standard deviation (SD), minimum (Min) and maximum (Max) of the number of positive cases of COVID-19 per 1,000 people in log at the city level by year and in total.

SI Table 4: Product category: The leftmost “Redefined category” is defined and used in this study. “Original category” was originally defined in the Yahoo! Shopping data. The original category had 17 items. However, because of the scarcity of the data, we dropped “gifts”, “housekeeping services”, “videos and music” and “books”. Therefore, 13 items are listed.

Redefined category	Original category	Representative product
Health products, foods, and medicine	Health goods	Sanitary goods
	Health foods	Supplements; Vitamin drinks
	Medicine	Drugs (for various symptoms)
Foods and household products	Foods	Breads; Cereal
	Household products	Detergent; Cleaning goods; Stationery
Nonessential products used mostly at home	Sweets and alcohol	Various snacks, fruits, and alcohol
	Consumer electronics	PCs; Cameras
	Furniture	Tables; Shelves
	Hand tools and car supplies	Set of tools; Paint
	Hobby supplies	Camp equipment; Climbing gear
	Goods for pets	Foods for dogs and cats; Cages
Nonessential mostly used outside the home	Cosmetics	Makeup goods; Skin-care goods
	Clothes	Clothes; Shoes

SI Table 5: Parallel Trend Checks Prior to the Treatment[4]

Dependent variables	(1) Purchases	(2) Buyers	(3) Purchases per buyer
LastHalf2019	-0.175**	-0.0976**	-0.0774**
	(0.00401)	(0.00296)	(0.00263)
FrequentSoE	0.710**	0.724**	-0.0142**
	(0.0620)	(0.0612)	(0.00510)
LastHalf2019XFrequentSoE	0.00427	0.00388	0.000387
	(0.00505)	(0.00382)	(0.00325)
Observations	368,060	368,060	368,060
R-squared	0.082	0.096	0.020

[4]This table shows results from the regression of the purchase amount (Column 1), the number of buyers (Column 2), and the purchase amount per buyer (Column 3). LastHalf2019 and FrequentSoE indicate the dummy variable for the last half of 2019 and the dummy variable for the prefectures that were in a state of emergency for longer than the median prefecture (47), respectively. LastHalf2019XFrequentSoE is the interaction term between the two. The standard errors, clustered at the city level, are in parentheses. ** p<0.01, and * p<0.05.

SI Table 6: Results of Equation (2) [5]

Dependent variables	(1) Purchases	(2) Buyers	(3) Purchases per buyer
Cases in wave 1	0.0200**	0.0257**	-0.00575
	(0.00524)	(0.00248)	(0.00437)
Cases in wave 2	0.0206**	0.0168**	0.00379*
	(0.00222)	(0.00111)	(0.00185)
Cases in wave 3	-0.00576**	-1.42e-05	-0.00575**
	(0.00201)	(0.00109)	(0.00167)
Cases in wave 4	-0.00365	-0.00203	-0.00162
	(0.00210)	(0.00120)	(0.00178)
Cases in wave 5	-0.00420	0.000239	-0.00444*
	(0.00238)	(0.00114)	(0.00214)
SoE in 04/2020	0.0738**	0.0554**	0.0185
	(0.0220)	(0.0115)	(0.0177)
SoE in 05/2020	-0.0172	-0.000918	-0.0162
	(0.0185)	(0.00977)	(0.0148)
SoE in 01/2021	0.0409**	0.0312**	0.00975
	(0.00615)	(0.00294)	(0.00534)
SoE in 02/2021	0.0404**	0.0191**	0.0213**
	(0.00576)	(0.00282)	(0.00474)
SoE in 03/2021	0.00226	0.00553	-0.00326
	(0.0105)	(0.00564)	(0.00849)
SoE in 04/2021	0.0556**	0.0339**	0.0217
	(0.0145)	(0.00583)	(0.0133)
SoE in 05/2021	0.0522**	0.0401**	0.0122*
	(0.00673)	(0.00337)	(0.00555)
SoE in 06/2021	0.0409**	0.0267**	0.0142*
	(0.00710)	(0.00349)	(0.00594)
SoE in 07/2021	0.0130	-0.00775	0.0207
	(0.0128)	(0.00569)	(0.0108)
SoE in 08/2021	0.0290**	0.00516	0.0238**
	(0.00787)	(0.00353)	(0.00693)
SoE in 09/2021	0.000685	-0.0105**	0.0112
	(0.00679)	(0.00338)	(0.00579)
Semi-SoE	0.00349	-0.00442*	0.00791*
	(0.00383)	(0.00203)	(0.00307)
SoE in 2020 before 4w	0.00710	0.00304	0.00407
	(0.00846)	(0.00337)	(0.00769)
SoE in 2020 before 3w	0.000734	-0.00776	0.00850
	(0.0119)	(0.00500)	(0.0101)
SoE in 2020 before 2w	0.0463**	0.0339**	0.0124
	(0.0156)	(0.00778)	(0.0121)
SoE in 2020 before 1w	0.0465**	0.0316**	0.0150
	(0.0177)	(0.00890)	(0.0142)
SoE in 20201 before 4w	0.00138	-0.00259	0.00397
	(0.00573)	(0.00266)	(0.00498)
SoE in 20201 before 3w	0.00765	0.00501	0.00264
	(0.00671)	(0.00325)	(0.00558)
SoE in 20201 before 2w	0.0229**	0.00960**	0.0134*
	(0.00644)	(0.00302)	(0.00530)
SoE in 20201 before 1w	0.0439**	0.0244**	0.0195**
	(0.00742)	(0.00365)	(0.00609)
Semi-SoE before 4w	-0.000108	-0.00107	0.000964
	(0.00522)	(0.00238)	(0.00451)
Semi-SoE before 3w	-0.00906	-0.00460	-0.00446
	(0.00511)	(0.00244)	(0.00442)
Semi-SoE before 2w	0.00351	-0.00396	0.00747
	(0.00507)	(0.00249)	(0.00445)
Semi-SoE before 1w	0.00798	0.00275	0.00523

	(0.00600)	(0.00277)	(0.00509)
SoE in 2020 after 1w	-0.0370*	-0.00875	-0.0282*
	(0.0146)	(0.00709)	(0.0121)
SoE in 2020 after 2w	-0.0217	-0.0127*	-0.00901
	(0.0123)	(0.00605)	(0.0104)
SoE in 2020 after 3w	-0.0148	-0.00205	-0.0127
	(0.00932)	(0.00473)	(0.00800)
SoE in 2020 after 4w	0.00224	0.00275	-0.000510
	(0.00777)	(0.00353)	(0.00680)
SoE in 20201 after 1w	-0.00200	-0.00696*	0.00496
	(0.00655)	(0.00336)	(0.00579)
SoE in 20201 after 2w	-0.00388	-0.0154**	0.0115*
	(0.00645)	(0.00291)	(0.00570)
SoE in 20201 after 3w	-0.0145*	-0.00863**	-0.00589
	(0.00628)	(0.00270)	(0.00567)
SoE in 20201 after 4w	-0.0183**	0.00277	-0.0210**
	(0.00662)	(0.00323)	(0.00572)
Semi-SoE after 1w	-0.00884	-0.00668	-0.00216
	(0.00736)	(0.00341)	(0.00635)
Semi-SoE after 2w	-0.000298	-0.000557	0.000260
	(0.00785)	(0.00315)	(0.00703)
Semi-SoE after 3w	0.00684	0.000996	0.00584
	(0.00716)	(0.00321)	(0.00621)
Semi-SoE after 4w	0.000203	-0.00135	0.00155
	(0.00671)	(0.00290)	(0.00614)
Neighbor Cases in wave 1	-0.00244	0.000437	-0.00288
	(0.00264)	(0.00141)	(0.00210)
Neighbor Cases in wave 2	0.00829**	0.00661**	0.00168
	(0.00167)	(0.000989)	(0.00130)
Neighbor Cases in wave 3	0.00612**	0.00525**	0.000876
	(0.00141)	(0.000834)	(0.00120)
Neighbor Cases in wave 4	0.00296	0.00498**	-0.00203
	(0.00160)	(0.000929)	(0.00128)
Neighbor Cases in wave 5	0.00367*	0.00792**	-0.00425**
	(0.00164)	(0.000950)	(0.00138)
Neighbor SoE in 04/2020	-0.00191	0.00538*	-0.00729
	(0.00492)	(0.00242)	(0.00437)
Neighbor SoE in 05/2020	-0.00606	0.00488*	-0.0109**
	(0.00469)	(0.00243)	(0.00394)
Neighbor SoE in 01/2021	0.00730*	0.00337*	0.00393
	(0.00295)	(0.00134)	(0.00254)
Neighbor SoE in 02/2021	0.00286	0.00179	0.00107
	(0.00286)	(0.00132)	(0.00238)
Neighbor SoE in 03/2021	-0.00890*	-0.00806**	-0.000839
	(0.00436)	(0.00234)	(0.00359)
Neighbor SoE in 04/2021	-0.000572	0.00920**	-0.00977
	(0.00747)	(0.00324)	(0.00671)
Neighbor SoE in 05/2021	0.0120**	0.00912**	0.00284
	(0.00338)	(0.00164)	(0.00276)
Neighbor SoE in 06/2021	0.0134**	0.00641**	0.00698*
	(0.00354)	(0.00173)	(0.00291)
Neighbor SoE in 07/2021	-0.00286	-0.0163**	0.0135
	(0.00994)	(0.00389)	(0.00863)
Neighbor SoE in 08/2021	-0.00314	-0.0105**	0.00733**
	(0.00282)	(0.00119)	(0.00251)
Neighbor SoE in 09/2021	0.00649*	-0.00491**	0.0114**
	(0.00256)	(0.000911)	(0.00243)
Neighbor Semi-SoE	-7.96e-05	-0.00557**	0.00549**
	(0.00162)	(0.000794)	(0.00138)
Observations	1,091,171	1,091,171	1,091,171
R-squared	0.912	0.980	0.216

[1] This table shows the results from the regression of the purchase amount (Column 1), the number of buyers (Column 2), and the purchase amount per buyer (Column 3) using Equation (2). "Cases" and "SoE" mean the number of COVID-19 cases and the declaration of a state of emergency, respectively. "Neighbor" indicates the number of cases or the declaration of an SoE in neighboring prefectures, whereas "before iw" and "after iw" represent i weeks before and after an SoE, respectively. The standard errors, clustered at the city level, are in parentheses. ** $p<0.01$, and * $p<0.05$.

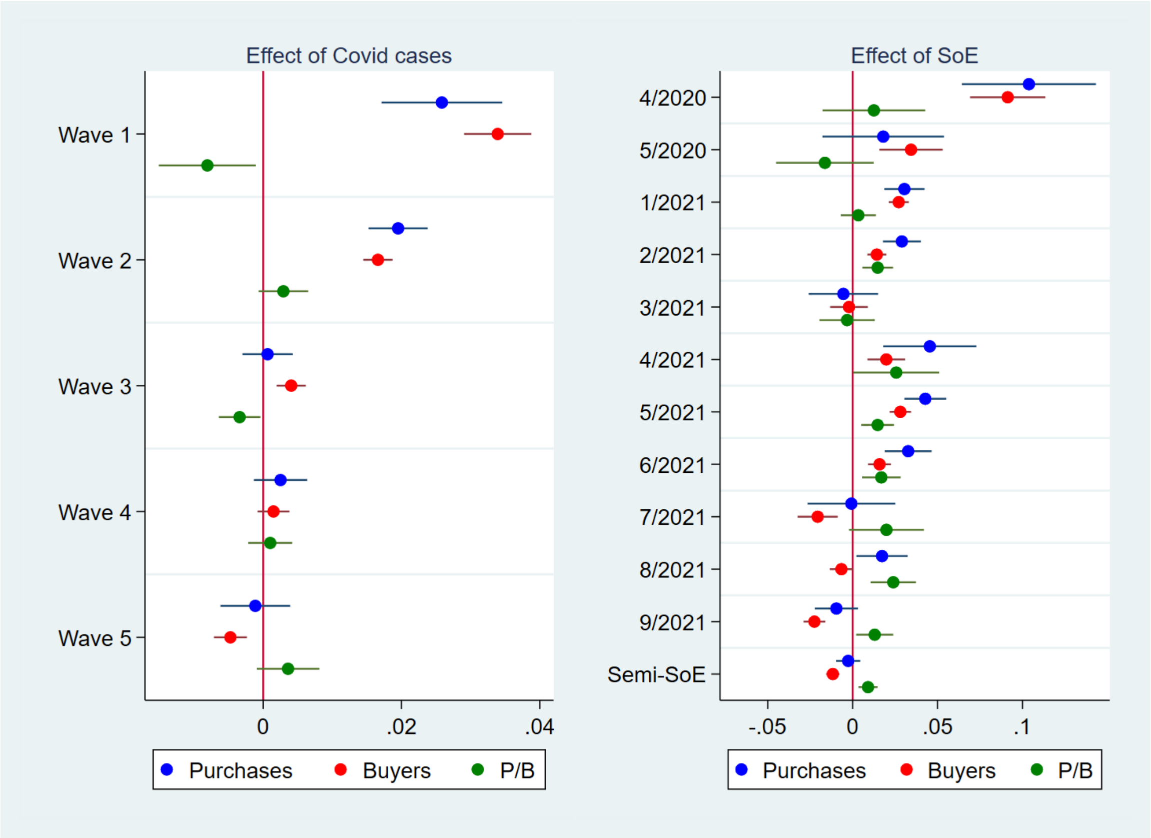

SI Figure 1: Effects of the Number of COVID-19 Cases and the Declaration of a State of Emergency on the Purchase Amount, the Number of Buyers, and the Purchase Amount per Buyer. This figure illustrates the results from the regressions of the purchase amount, the number of buyers, and the purchase amount per buyer in logs at the city-date level. The left panel shows the point estimates and 95% confidence intervals of the effect of the number of COVID-19 cases per 1,000 persons averaged over one week on the purchase amount (blue), the number of buyers (red), and the purchase amount per buyer (green) in the five waves of COVID-19 in the sample period. The right panel shows the coefficients and 95% confidence intervals of the declaration of a state of emergency (SoE), separated into the months of the sample period and a semi-SoE, which is less restrictive than an SoE. The number of COVID-19 cases and the declaration of an SoE are given at the prefecture-date level. We take a log of the number of COVID-19 cases per 1,000 persons plus 0.001 to incorporate possible nonlinear relationships. In all the regressions, the other independent variables are the number of neighboring prefectures in an SoE; the dummy variables for 1-4 weeks before and after the declaration of an SoE and a semi-SoE; and the city, date, and day-of-the-week fixed effects. The standard errors are clustered at the city level. N=1,091,171.

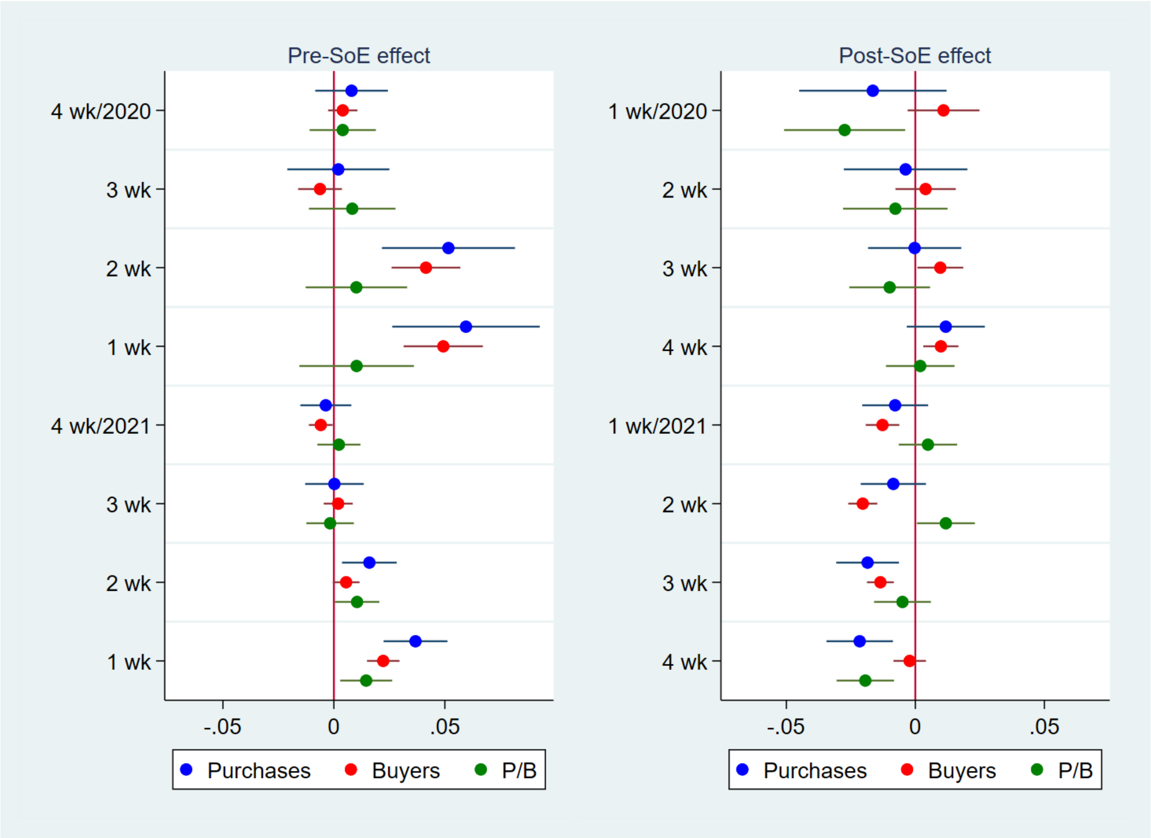

SI Figure 2: Pre- and Posttreatment Effects of the Declaration of a State of Emergency on the Purchase Amount, the Number of Buyers, and the Purchase Amount per Buyer. This figure illustrates the results of the regressions of the purchase amount, the number of buyers, and the purchase amount per buyer in logs at the city-date level. The left panel shows the point estimates and 95% confidence intervals of the dummies for 1-4 weeks before the declaration of a state of emergency (SoE) on the purchase amount (blue), the number of buyers (red), and the purchase amount per buyer (green). The right panel shows those of the dummies for 1-4 weeks after the end of an SoE. The dummies for before and after an SoE are given at the prefecture-date level. For example, "4 wk/2020" on the x-axis of the left panel means 4 weeks before an SoE in 2020, whereas "1wk/2020" in the right panel means 1 week after an SoE in 2020. In all the regressions, the other independent variables are the number of COVID-19 cases per 1,000 persons; the dummies of an SoE; the numbers of neighboring prefectures in an SoE; and the city, date, and day-of-the-week fixed effects. The standard errors are clustered at the city level. N=1,091,171.

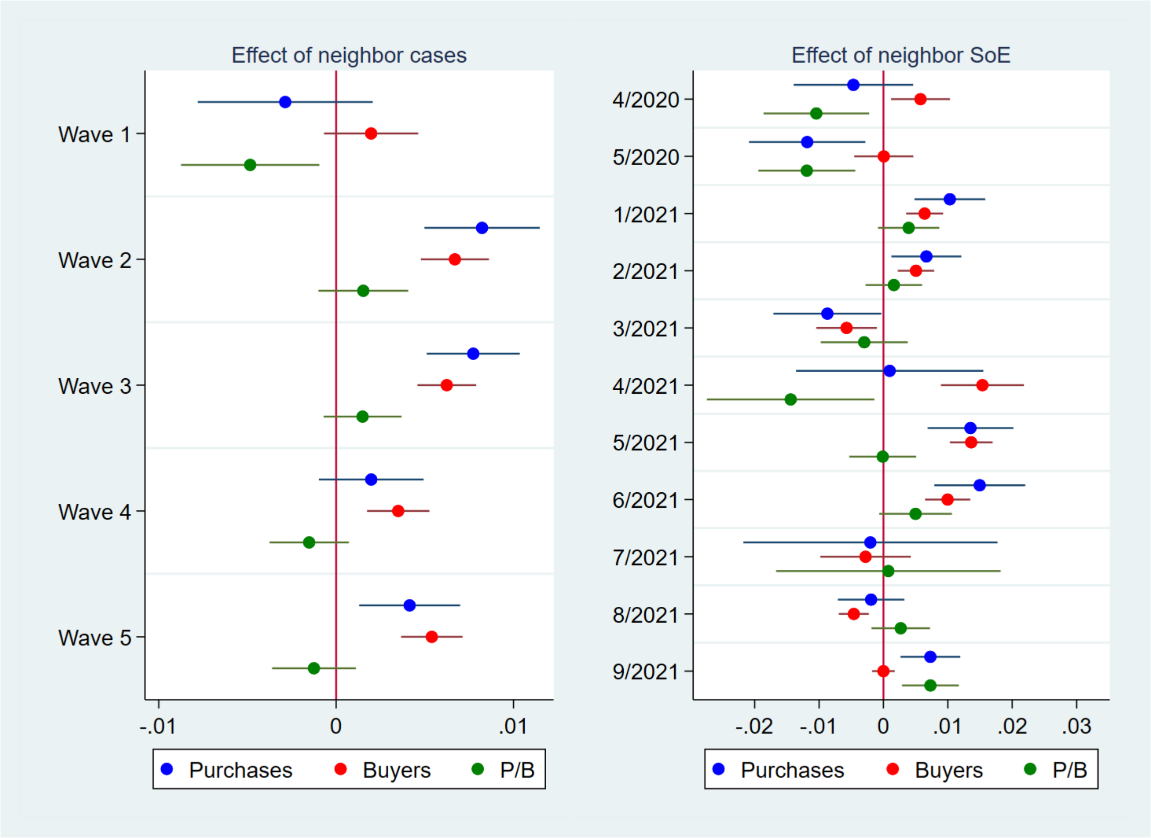

SI Figure 3: Effects of the Declaration of a State of Emergency in Neighboring Prefectures on the Purchase Amount, the Number of Buyers, and the Purchase Amount per Buyer. This figure illustrates the point estimates and 95% confidence intervals of the effect of the number of prefectures that were in a state of emergency (SoE) and shared any border with the focal prefecture in different months on the purchase amount (blue), the number of unique buyers (red), and the purchase amount per unique buyer (green) in the logs. In all the regressions, the other independent variables are the number of COVID-19 cases per 1,000; the dummies of an SoE; the dummies for before and after an SoE; the number of neighboring prefectures in an SoE; and the city, date, and day-of-the-week fixed effects. The standard errors are clustered at the city level. N=1,091,171.
